# Four-Year Longitudinal Phenotyping of Post-Acute Sequelae of SARS-CoV-2 Infection (PASC): A Single-Centre Cohort Study

**DOI:** 10.3390/jcm15197487

**Published:** 2026-09-26

**Authors:** Anna Teréz Sárközi, Ilona Tornyi, Ildikó Horváth

**Affiliations:** 1 Department of Pulmonology, Faculty of Medicine, University of Debrecen, 4032 Debrecen, Hungary; sarkozi.anna@med.unideb.hu (A.T.S.); horvath.ildiko@med.unideb.hu (I.H.); 2 Doctoral School of Medical Sciences, University of Debrecen, 4032 Debrecen, Hungary; 3 National Koranyi Institute of Pulmonology, 1122 Budapest, Hungary

**Keywords:** cluster analysis, long COVID, phenotypes, post-acute sequelae of SARS-CoV-2 infection (PASC), longitudinal cohort study, pulmonary rehabilitation, symptom persistence

## Abstract

**Background**: Although an increasing number of studies have reported 2- and 3-year outcomes of post-acute sequelae of SARS-CoV-2 infection (PASC), the long-term symptom patterns of and their clinical phenotypes beyond four years remains unknown. We aimed to characterize 48-month clinical outcomes and symptom profiles using hierarchical cluster analysis. **Methods**: We conducted a longitudinal cohort study of 79 patients with persistent PASC enrolled between March 2021 and March 2022. Of these, 77 patients completed the 48-month follow-up. Symptoms were assessed at 11 and 48 months post-infection. Hierarchical cluster analysis using Jaccard distance and Ward’s linkage identified symptom-based phenotypes at 48 months. Cluster membership was subsequently mapped back to 11-month symptom profiles to evaluate longitudinal trajectories. **Results**: Of the 77 patients completing the 48-month follow-up, symptom profiles at 48 months could be descriptively characterized into four exploratory, symptom-based profiles, which differed from the broader symptom patterns observed at month 11: Cluster 1 (cardiorespiratory and neurosensory symptoms; 16.9%, *n* = 13), characterized by persistent chest discomfort and palpitations; Cluster 2 (musculoskeletal/pain-predominant; 33.8%, *n* = 26), characterized by persistent myalgia and arthralgia. Cluster 3 (neurocognitive and mixed multisystem profile; 23.4%, *n* = 18), characterized by persistent cognitive impairment and musculoskeletal pain and Cluster 4 (minimal residual symptom burden; 26.0%, *n* = 20), demonstrating the lowest overall residual symptom burden at 48 months. **Conclusions**: At 48 months, PASC symptoms showed substantial heterogeneity, with four exploratory symptom-based profiles. In our cohort, over one-quarter of patients had the lowest observed residual symptom burden at 48 months; neurocognitive and musculoskeletal symptoms remained prominent in specific profiles, supporting the need for targeted multidisciplinary post-acute care.

## 1. Introduction

A substantial proportion of COVID-19 survivors develop persistent or de novo multisystem symptoms extending well beyond acute infection. The condition recognized as post-acute sequelae of SARS-CoV-2 infection (PASC), and commonly referred to as Long COVID or chronic/post-COVID-19, represents a complex, multi-systemic condition [1,2,3,4,5]. According to the current official World Health Organization (WHO) clinical case definition, PASC is characterized by symptoms occurring in individuals with a history of probable or confirmed SARS-CoV-2 infection, usually three months from the onset of COVID-19, lasting for at least two months, and not being explained by an alternative diagnosis [2].

Early observations demonstrated that post-acute symptoms (subacute/ongoing COVID) could be detected in the majority of patients during the first three months after acute infection [5]. Further observational studies assessing long-term health burden and symptom remission/persistence over longer time have shown widely variable presentations and disease trajectories all around the world [6,7,8,9]. Huang L. et al. pointed out that despite overall improvement, the burden of symptomatic sequelae remains fairly high for two years after hospitalization due to COVID-19 [6]. Cai M. et al. found reduction in risk of death and morbidity over time; however, significantly elevated risk levels remained even three years after hospitalization in a different cohort [7]. In another large longitudinal study, it was shown that overall long COVID symptoms were mild to moderate at 3-year follow-up [8]. In a four-year follow-up after hospital discharge, reduction in symptoms was reported over time, but reinfection was detected in 36.1% of patients [9]. The study also showed that reinfections and older age were associated with symptom persistence. Similar findings were found in a Middle Eastern cohort followed up for a median of 24 months, demonstrating that delayed recovery was associated with reinfection and hospitalization [10]. 

Long-COVID trajectories over a 15-month observational period were determined using a research index threshold for Long COVID in the RECOVER Adult cohort, and eight distinct profiles were described based on symptom burden [11,12]. Using a numerical index to characterize the severity and progress of PASC as an overall indicator seems to be a good overarching approach; however, it does not allow deeper understanding of the ongoing processes. Due to the clinical complexity of PASC, traditional organ-specific study designs are also inadequate [13]. Data-driven multivariable approaches, particularly hierarchical cluster analysis, have long been used in respiratory medicine to identify clinically meaningful disease phenotypes [14] and have more recently been applied to post-acute sequelae of SARS-CoV-2 infection [15,16,17,18]. However, existing cluster analyses have largely been conducted cross-sectionally at single post-infection time points. 

We investigated the 48-month clinical outcomes and phenotypes of persistent PASC in an outpatient cohort enrolled between 2021 and 2022. Utilizing hierarchical cluster analysis on endpoint symptom profiles, we evaluated clinical phenotypes and examined whether these late-stage profiles correlate with early clinical patterns at 11-month follow-up.

## 2. Materials and Methods

### 2.1. Study Design

We conducted a retrospective, longitudinal cohort study within the Post-COVID Outpatient Care Network of the Clinical Centre of the University of Debrecen (DEKK), Debrecen, Hungary. Patients were enrolled between 1 March 2021 and 1 March 2022. The study was conducted in accordance with the principles of the Declaration of Helsinki and received approval from the Regional and Health Sciences Research Ethics Committee (DEOEC RKEB/IKEB 5959-2022; ETT TUKEB BM/29856-1/2024).

To ensure homogeneous study population, we applied a rigorous, multi-step inclusion protocol (Figure 1). Only patients with documented, persistent PASC symptoms were enrolled. Hospitalization due to acute COVID-19 was an additional inclusion criterion; therefore, all patients included in the study had been hospitalized during the acute phase of SARS-CoV-2 infection. Following the acute infection, all participants underwent a comprehensive baseline evaluation comprising detailed laboratory biomarker testing, pulmonary function testing, and chest computed tomography (CT). An additional inclusion criterion required that each patient attended the Post-COVID Clinic at the DEKK Pulmonology Department on at least two occasions.

Symptoms were assessed using a standardized, structured symptom questionnaire developed for the post-COVID outpatient care network. All symptom assessments were based exclusively on patient-reported responses. The same questionnaire, symptom definitions, and questioning methodology were used at both the initial 11-month and final 48-month follow-up assessments during consultations. Only patients with fully completed symptom questionnaires were included in the study.

### 2.2. Study Cohort Characterization and Follow-Up

During the screening phase, records from 238 patients referred by their primary care physicians were reviewed. The first outpatient assessment took place at a median of 128 days (IQR: 99.25–180.75) following acute infection. After application of inclusion and exclusion criteria, the final study cohort comprised 79 patients (Figure 1 and Figure 2).

Over the 48-month follow-up period, two deaths occurred (attributable to recurrence of lung cancer and reactivation of chronic lymphocytic leukemia, respectively). Both conditions had been diagnosed before the acute COVID-19 event. Accordingly, 77 patients completed the full follow-up and formed the basis for the endpoint cluster analysis. The overall retention rate was 97.47% (attrition rate: 2.53%). COVID-19 severity was classified according to the Diagnosis and Treatment Protocol for Novel Coronavirus Pneumonia (Trial Version 7) [19].

### 2.3. Statistical Analysis

All statistical analyses were performed using the R statistical environment, version 4.5.2 (R Foundation for Statistical Computing, Vienna, Austria) within RStudio IDE version 2025.05.0 + 496. The normality of continuous variables was assessed using the Shapiro–Wilk test. Normally distributed variables are reported as mean ± standard deviation (SD); non-normally distributed variables are reported as median and interquartile range (IQR). Categorical variables are described as absolute frequencies and percentages [n (%)]. To evaluate the longitudinal dynamics of individual symptoms within each 48-month endpoint-defined cluster between the 11-month and 48-month assessments, exact McNemar’s tests for paired nominal data was performed. For longitudinal symptom analysis, persistence was defined as the proportion of participants who reported a given symptom at baseline and continued to report the symptom at follow-up (1 → 1). The denominator for each persistence proportion was the number of participants who were symptomatic at baseline (1 → 0 + 1 → 1). Exact 95% confidence intervals (CIs) for persistence proportions were calculated using the exact binomial method. A *p*-value of less than 0.05 was considered statistically significant for a change in symptom prevalence (improvement or worsening), while a non-significant *p*-value (*p* ≥ 0.05) was interpreted as an absence of statistically detectable change in symptom prevalence, rather than as evidence of symptom fixation or chronicity. To account for multiple comparisons, *p*-values from the McNemar tests across the four clusters were adjusted using the Benjamini–Hochberg procedure, with a false discovery rate (FDR) threshold of 0.05.

### 2.4. Cluster Structure Development and Phenotyping

Cluster analysis was performed on a binary symptom matrix recorded at the 48-month endpoint (0 = symptom absent; 1 = symptom present), reflecting the long-term clinical status of each participant. Phenotypes were accordingly defined based on endpoint symptom patterns. Binary (Jaccard) dissimilarities were calculated using the base R dist() function with method = “binary”, and hierarchical clustering was performed using the hclust() function from the R stats package with the Ward.D2 method. The number of clusters (*n* = 4) was predefined based on our previous study [17]. Although the same number of clusters was used, cluster membership was determined independently from our previous study. Once cluster assignments were established, they were treated as fixed phenotype groups and retrospectively applied to the symptom profiles of the same patients recorded 11 months post-infection, enabling longitudinal trajectory analysis.

Subgroup-specific temporal symptom dynamics and clinical trajectories were visualized using radar charts, which also permitted direct comparison of symptom prevalence (%) between the 11- and 48-month assessments.

## 3. Results

**Forty-Eight-Month Follow-Up: Endpoint Symptom Profiles**

By 48 months post-infection assessment, the clinical presentation of the cohort (*n* = 77) could be descriptively characterized into four symptom-based profiles, which differed in their predominant symptom patterns (Figure 3A). These profiles were defined a priori according to the four-cluster framework used in the analysis and should be interpreted as exploratory, descriptive phenotypic patterns rather than as formally validated clinical subtypes.

The four endpoint subgroups were defined as follows:

Cluster 1—Cardiorespiratory and neurosensory residual phenotype (*n* = 13): Respiratory symptoms decreased substantially between the two assessments, although cough and shortness of breath remained present in a subset of patients. Persistent symptoms were predominantly limited to chest discomfort and palpitations, with hearing impairment representing the principal localized neurosensory residual manifestation.

Cluster 2—Isolated musculoskeletal/pain-predominant phenotype (*n* = 26): This subgroup was characterized by residual musculoskeletal symptoms—specifically myalgia and arthralgia—in the absence of neurocognitive or respiratory complaints at the 48-month assessment.

Cluster 3—Neurocognitive and mixed multisystem phenotype (*n* = 18): This subgroup showed the highest observed residual symptom burden within the cohort, exhibiting the most pronounced clustering of cognitive impairment and arthralgia. Central nervous system manifestations were accompanied by mixed cardiorespiratory symptoms.

Cluster 4—Minimal residual symptom burden phenotype (*n* = 20): This cluster constituted the largest contiguous symptom-free region on the heatmap. Previously prevalent subjective symptoms—sore throat, dizziness, and tinnitus—had largely resolved completely. Patients in this subgroup indicated a markedly reduced symptom burden at the 48-month assessment.

Across the full study cohort, sensory dysfunctions—including anosmia and ageusia—had almost completely resolved by the end of the 48-month follow-up.

Following characterization of the 48-month endpoint symptom profiles, we retrospectively examined the baseline demographic characteristics, relevant comorbidities, and smoking history of each subgroup (Table 1). The characteristics and severity of the acute COVID-19 infection are presented in Table 2.

**Eleven-month assessment: baseline symptom patterns according to endpoint cluster assignment**

At 11 months post-infection, the cohort (*n* = 77) exhibited extensive multisystem involvement, characterized by a diffuse, high-density distribution of symptoms across nearly all assessed organ systems (Figure 3B). Retrospective projection of the 48-month cluster assignments onto these early profiles revealed distinct, baseline symptom patterns:

Cluster 1: Heatmap analysis revealed horizontally continuous regions of high symptom density. Cardiorespiratory manifestations (dyspnoea, chest discomfort, and palpitations), neurocognitive symptoms (headache and cognitive dysfunction), and systemic musculoskeletal complaints were concurrently present at high prevalence.

Cluster 2: During the early phase, musculoskeletal complaints were accompanied by substantial neurocognitive symptoms. These additional manifestations resolved progressively over time, resulting in a 48-month profile characterized predominantly by myalgia and arthralgia.

Cluster 3: This subgroup exhibited high symptom density across major clinical domains, including cough, dyspnoea, cognitive impairment, and headache. However, sensory dysfunctions (anosmia, ageusia, and hearing impairment) and certain peripheral neurological complaints displayed a more fragmented, band-like pattern of lower density within this cluster.

Cluster 4: Although cognitive dysfunction and musculoskeletal pain persisted for 11 months, accompanying subjective symptoms—including sore throat, dizziness, and tinnitus—were already markedly less frequent in this subgroup, presaging substantial long-term clinical improvement.

Overall, at 11 months post-infection, symptom profiles had not yet resolved into organ-specific syndromes. Subgroup differentiation at this stage was driven primarily by overall symptom burden and the extent of multi-domain symptom co-occurrence.

**Cluster-specific changes in symptom prevalence between the 11- and 48-month assessments**

Whereas the heatmaps illustrate aggregate differences in symptom prevalence across the full cohort, a more detailed description of changes in symptom prevalence between the two assessment points requires individual longitudinal analysis of each identified cluster. The radar charts (Figure 4) depict the distinct temporal changes in symptom prevalence between the 11- and 48-month assessments for each clinical phenotype. Detailed symptom prevalence at 11 and 48 months for each cluster is provided in Supplementary Table S1.

Cluster 1: The 11-month profile was characterized by a broad PASC burden spanning nearly the full symptom spectrum, with respiratory symptoms (cough and dyspnoea), neurocognitive complaints (cognitive dysfunction and headache), and cardiovascular symptoms (chest discomfort and palpitations) all exceeding 75% prevalence. By 48 months, the clinical profile had undergone marked restructuring. Primary pulmonary and upper respiratory symptoms were substantially less prevalent at 48 months, whereas chest discomfort and palpitations remained among the more prevalent residual symptoms at the 48-month assessment. This profile was characterized by residual cognitive dysfunction, peripheral neuropathy, and hearing impairment.

Cluster 2: The radar chart illustrates a characteristic change in symptom prevalence. While neurocognitive and cardiopulmonary comorbid symptoms were less prevalent by 48 months, myalgia and arthralgia persisted as an isolated syndrome, with prevalence exceeding 75%.

Cluster 3: This subgroup showed a relatively high residual symptom burden at the 48-month assessment, particularly for cognitive, peripheral neurological, and musculoskeletal symptoms. Despite partial improvement in respiratory symptoms by 48 months, cognitive impairment, peripheral neuropathy, and accompanying musculoskeletal symptoms (myalgia and arthralgia) remained at prevalences exceeding 75%.

Cluster 4: This subgroup showed the largest reduction in observed symptom prevalence between the 11- and 48-month assessments. The moderate multisystem burden present at 11 months had resolved almost entirely by 48 months, with residual symptom prevalence falling below 5%, indicating a substantially reduced symptom burden at the 48-month assessment.

Collectively, the heatmaps and radar charts describe differences in symptom prevalence and residual symptom burden between the 11- and 48-month assessments across the four predefined clusters. They also identify the predefined subgroups with the highest observed residual symptom burden at 48 months.

**Statistical confirmation of longitudinal symptom dynamics: Intra-cluster McNemar analysis**

McNemar tests performed within each cluster confirmed that prevalence differed between 11 and 48 months after SARS-CoV-2 infection (Table 3). Instead, symptom prevalence differed between the two assessment points, with the magnitude and direction of change varying across the four predefined clusters. After Benjamini–Hochberg correction for multiple comparisons, most statistically significant findings remained significant, although only a few associations lost statistical significance; these findings should be interpreted cautiously.

Cluster 1: Respiratory symptoms resolved progressively, whereas cardiovascular manifestations showed no statistically significant change. While cough (*p* = 0.0200) decreased significantly between months 11 and 48, chest discomfort (*p* = 0.6154) and palpitations (*p* = 0.6154) remained unchanged, indicating that cardiovascular symptoms constituted an important residual symptom burden in this cluster at 48 months.

Cluster 2: General and upper respiratory symptoms showed improvement, with significant reduction in cough, shortness of breath, sore throat, chest discomfort (*p* < 0.001) and headache (*p* < 0.001). Cognitive impairment (*p* < 0.001) and peripheral neuropathy (*p* = 0.0357) also improved. In contrast, joint and muscle pain showed no statistically detectable change between the two assessment points (*p* = 1.000). 

Cluster 3: Central nervous system impairment showed no statistically detectable change in prevalence between the two assessment points. Cognitive impairment showed no statistically detectable change. Cough, shortness of breath, chest discomfort, and palpitation improved significantly (all *p* < 0.05). Musculoskeletal symptoms remained stable. Cognitive and musculoskeletal symptoms constituted important residual symptom domains at the 48-month assessment.

Cluster 4: McNemar analysis demonstrated widespread, progressive resolution in symptom prevalence across multiple symptom domains. General inflammatory and upper respiratory symptoms decreased significantly, including sore throat (*p* = 0.0122) and headache (*p* = 0.0026). Respiratory and cardiovascular manifestations also improved substantially, with significant reductions in cough (*p* = 0.0122), shortness of breath (*p* < 0.001), chest discomfort (*p* < 0.001), and palpitations (*p* = 0.0131). Cognitive complaints (*p* = 0.0078) and joint pain (*p* = 0.0078) also decreased. This cluster showed the largest overall reduction in observed symptom prevalence between the two assessment points.

## 4. Discussion

Our findings confirm the fundamental heterogeneity of PASC at 48 months and identify four exploratory symptom-based profiles with different patterns of residual symptom burden. The observed changes describe differences between the 11- and 48-month assessments rather than continuous trajectories over the intervening period.

The main strength of this study is the 48-month follow-up horizon. While 2- and 3-year data suggest progressive symptom attenuation [8], our findings indicate that some symptoms remained prevalent at the 48-month assessment in specific clusters. At the 48-month assessment, symptom burden was much lower than at previous follow-up time points for most survivors. Even if our results regarding the prevalence of symptoms present after four years post-COVID cannot be compared directly with those of other studies due to different study settings, it points in the same direction: symptom burden decreased substantially overall; however, it remained fairly high in some patients. Large-scale international programs, including the US RECOVER initiative and the UK PHOSP-COVID study, have faced significant methodological challenges due to high attrition rates during multi-year evaluations [5,20]. Our study achieved a high retention rate of 97.47% after the final longitudinal cohort was established. However, a critical methodological distinction must be made between post-enrollment and pre-enrollment exclusions in our study. During the follow-up period, two deaths occurred, both related to pre-existing conditions diagnosed before the acute COVID-19 event. However, the extent to which COVID-19 may have contributed to the reactivation or subsequent deterioration of these conditions cannot be determined from our data, although the broader potential association between viral infections and oncological outcomes warrants continued vigilance [21].

**Clinical phenotypes and cluster-specific findings**

The present analysis differs substantially from our previous study despite using the same underlying cohort and identifying four symptom-based groups [17]. In our previous study, patients were classified according to the presence or absence of broad clinical symptom domains, whereas the present study applies hierarchical clustering to individual symptoms, enabling a more detailed and data-driven characterization of symptom profiles and their differences between the two assessment time points.

In our cohort, respiratory symptoms had largely resolved in nearly all patients by 48 months. This observation is in line with the results of others showing relatively fast and substantial lung function improvement after COVID [22]. At the same time, it is important to note that some patients had remaining respiratory symptoms even after 4 years following the acute infection. A similar observation was described by Han X. et al. 3 years after COVID-19 [23]. Epithelial repair programs are variable in the lung and their dysfunction has been suggested as an important mechanism responsible for the lack of symptom resolution [24]. This area warrants further studies to assess their association with clinical phenotypes in PASC.

The clinical profile of Cluster 1 showed a difference between pulmonary and cardiovascular residual symptom persistence at the 48-month assessment. This observation might urge further research to understand organ-specific differences in recovery. 

The long-term clinical profile of the Cluster 2 subgroup was defined by persistent musculoskeletal complaints, with joint pain showing the greatest persistence and muscle pain demonstrating no significant overall change at 48 months. Despite the progressive resolution of general inflammatory and upper respiratory symptoms, musculoskeletal complaints persisted. Baseline characterization of this subgroup showed that metabolic and allergic comorbidities, including diabetes mellitus and bronchial asthma, were present among patients in this cluster. Previous studies have proposed low-grade inflammation and altered tissue repair as potential mechanisms underlying persistent musculoskeletal symptoms in PASC [16,25]. In Cluster 3, persistent neurocognitive dysfunction remained unchanged throughout the 48-month assessment, despite significant improvement of cardiorespiratory symptoms. Our findings are consistent with previous studies pointing out long-term neurocognitive impairment years after COVID-19 [6,20,26]. Its pathomechanism is unclear despite the proposed contribution of residual neuro- (and systemic) inflammation, microvascular dysfunction, and prolonged hypoxia. As observed in our and other long-term hospitalization cohorts [6,26], the persistence of neurocognitive symptoms may identify patients who could benefit from sustained, targeted rehabilitation. Furthermore, it demonstrates the great need for timely, structured follow-up of these functions, and further research to develop appropriate treatment options. 

Cluster 4 represented the profile with the lowest observed residual symptom burden at 48 months. Despite mild heterogeneous symptoms during the first year following infection, the lowest residual symptom burden was observed in this cluster at 48 months follow-up. The lower symptom burden observed at 48 months may be compatible with previously proposed mechanisms involving resolution of immune activation and post-infectious inflammatory processes. Our findings are consistent with observations from large international cohorts, such as PHOSP-COVID and RECOVER, reporting substantial symptom improvement over extended follow-up periods [11,20]. However, because our study included only two symptom assessments, we cannot determine whether the low symptom burden observed at 48 months was continuously maintained throughout the intervening period or whether intermittent relapse or symptom fluctuation occurred. A defining feature of chronic PASC is the dissociation between persistent symptoms and normal routine investigations, including inflammatory markers and chest HRCT [4,13,18]. Relying solely on objective testing would misclassify symptomatic patients as recovered, highlighting the need for symptom-based phenotyping, especially in combination with clinical and functional outcomes [27].

**Limitations**

Several methodological limitations should be considered when interpreting these findings.

The study population comprised patients who attended our Post-COVID Outpatient Care Network during the early phase of the pandemic (2021–2022). In the absence of virological sequencing, precise identification of the infecting variant was not possible. Based on the timing of enrolment, patients were most likely infected with the ancestral Wuhan strain or the Alpha and Delta variants. The study period may have encompassed different pandemic waves, introducing potential heterogeneity in the infecting SARS-CoV-2 variant. This temporal heterogeneity should be considered when interpreting the long-term findings. Our results primarily reflect the long-term sequelae of early SARS-CoV-2 strains and should not be extrapolated to Omicron subvariants or subsequent variants.

Incomplete vaccination data precluded assessment of the impact of vaccination status on long-term outcomes. The absence of an uninfected control group limits causal inference; it cannot be entirely excluded that ageing or other environmental factors contributed to the changes observed over the 48-month follow-up.

The relatively small sample size (*n* = 77) and single-center design limit the generalizability of our findings to the broader population. The sample size of this study is insufficient to detect smaller effect sizes at the subgroup level, and therefore, findings from comparisons between clusters should be interpreted with caution. The study cohort consisted of ethnically homogeneous, white Caucasian patients, constraining extrapolation to patient groups from different geographical regions.

Incomplete baseline records among patients who dropped out before cohort finalization prevented a direct comparison between included and excluded patients, which may introduce the potential for selection bias.

BMI data were not available for all participants; therefore, it was not included in the analysis. Systematic monitoring of SARS-CoV-2 reinfections was not performed, as subsequent infections were not PCR-confirmed but identified through self-report and/or rapid antigen testing.

The number of clusters was predefined based on our previous study [17]; therefore, alternative clustering solutions cannot be formally excluded. The robustness of the identified clusters was not assessed using an alternative clustering strategy or an internal cluster-validity or stability assessment. Although the identified phenotypes formed clinically coherent subgroups, the cluster structure has not been independently validated in an external cohort. Our results should accordingly be regarded as primarily hypothesis-generating, and replication in larger, multicenter studies will be required to confirm reproducibility. Following Benjamini–Hochberg correction for multiple comparisons, some of the previously significant findings no longer remained statistically significant and should therefore be interpreted with caution. Symptom assessment relied on self-reported questionnaires. The symptom questionnaire did not include a graded severity scale for individual symptoms; therefore, symptoms were recorded as present or absent and subsequently coded as binary variables for the clustering analysis. This approach may have resulted in a loss of information regarding differences in symptom severity and the magnitude of change in individual symptoms over time. Fatigue was assessed as part of the quality-of-life questionnaire at the 11-month assessment; however, this questionnaire was not completed consistently at the 48-month follow-up. Therefore, fatigue was not included in the longitudinal clustering analysis.

Routine laboratory data and chest CT imaging were available for a subset of participants but were excluded from statistical analyses owing to the absence of standardized measurement protocols and substantial data heterogeneity. Laboratory parameters collected during the initial phase also provided limited diagnostic specificity for longitudinal phenotyping. This methodological decision is consistent with evidence from international studies demonstrating that standard inflammatory and metabolic biomarkers do not correlate with PASC symptom severity or long-term persistence [25].

## 5. Conclusions

Our 48-month follow-up demonstrates that PASC does not represent a uniform clinical entity, but rather a heterogeneous spectrum of long-term symptom patterns with differing levels of residual symptom burden at the 48-month assessment. In our cohort, just over one-quarter of patients who completed the 48-month follow-up had the lowest observed residual symptom burden, while neurocognitive and musculoskeletal symptoms remained prominent in specific symptom-based profiles at 48 months. The long-term burden of these symptoms provides a rationale for maintaining targeted multidisciplinary rehabilitation capacity—encompassing neurological, cognitive, and rheumatological care—beyond pulmonology alone. Furthermore, these findings call for preemptive multidisciplinary follow-up and further research for better treatment options for these patients. These findings are exploratory and require validation in larger studies with repeated assessments over time.

## Figures and Tables

**Figure 1:**
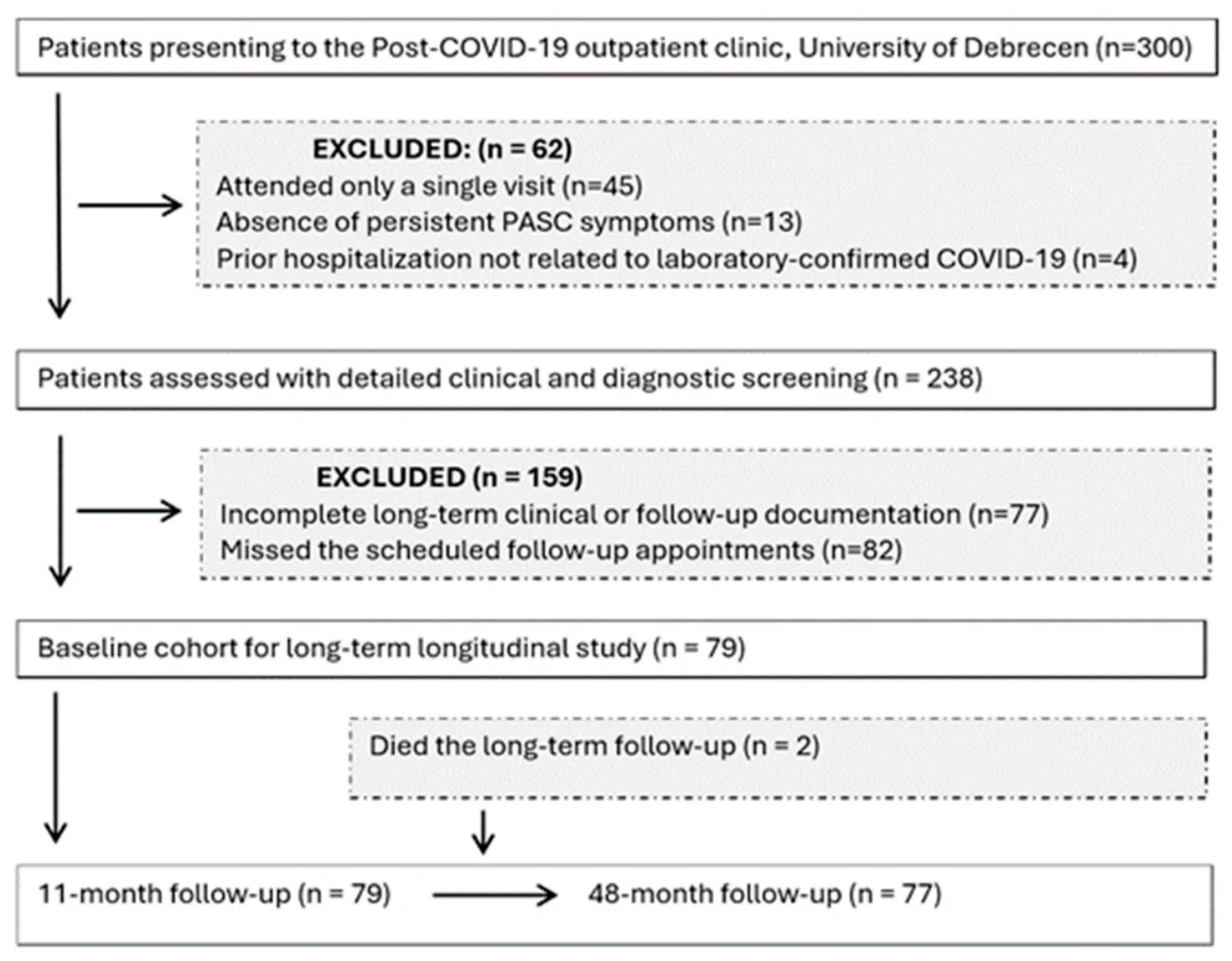
Flowchart of patient selection and long-term (11- and 48-month) follow-up.

**Figure 2:**
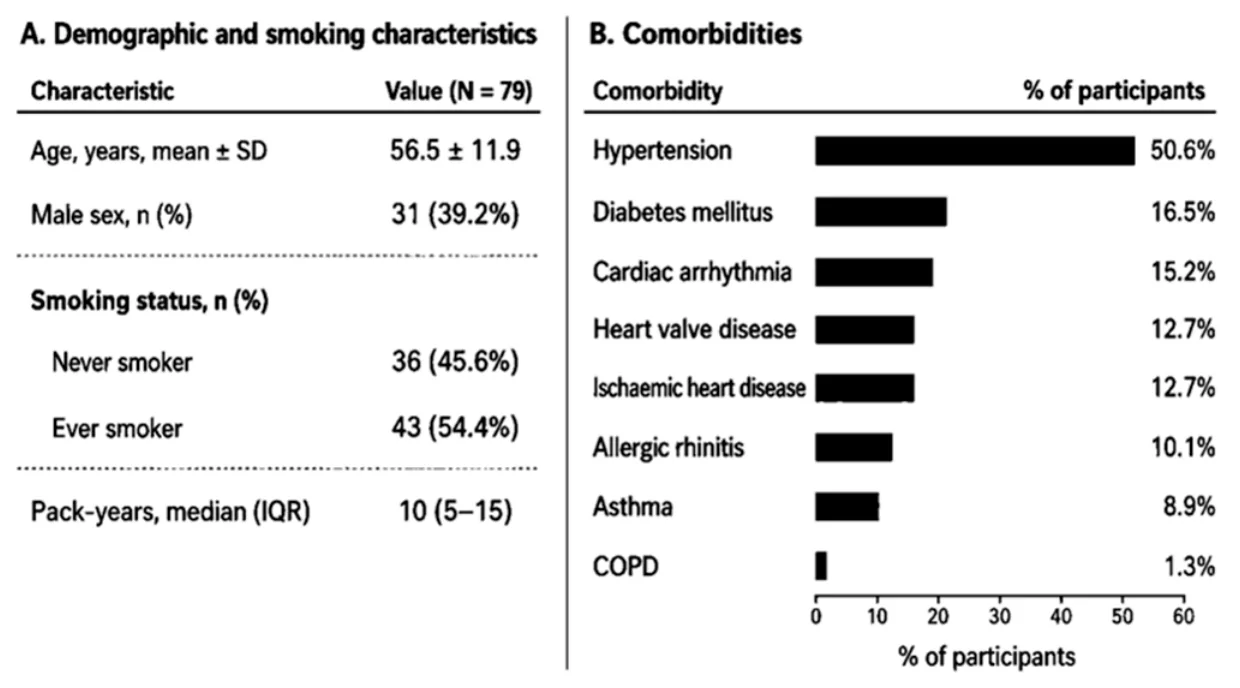
Baseline demographic and clinical characteristics of the study cohort (*n* = 79). (**A**) Demographic data and tobacco exposure metrics. (**B**) Prevalence of major baseline comorbidities Data are presented as n (%), mean ± SD, or median (IQR). COPD: chronic obstructive pulmonary disease; IQR: interquartile range.

**Figure 3:**
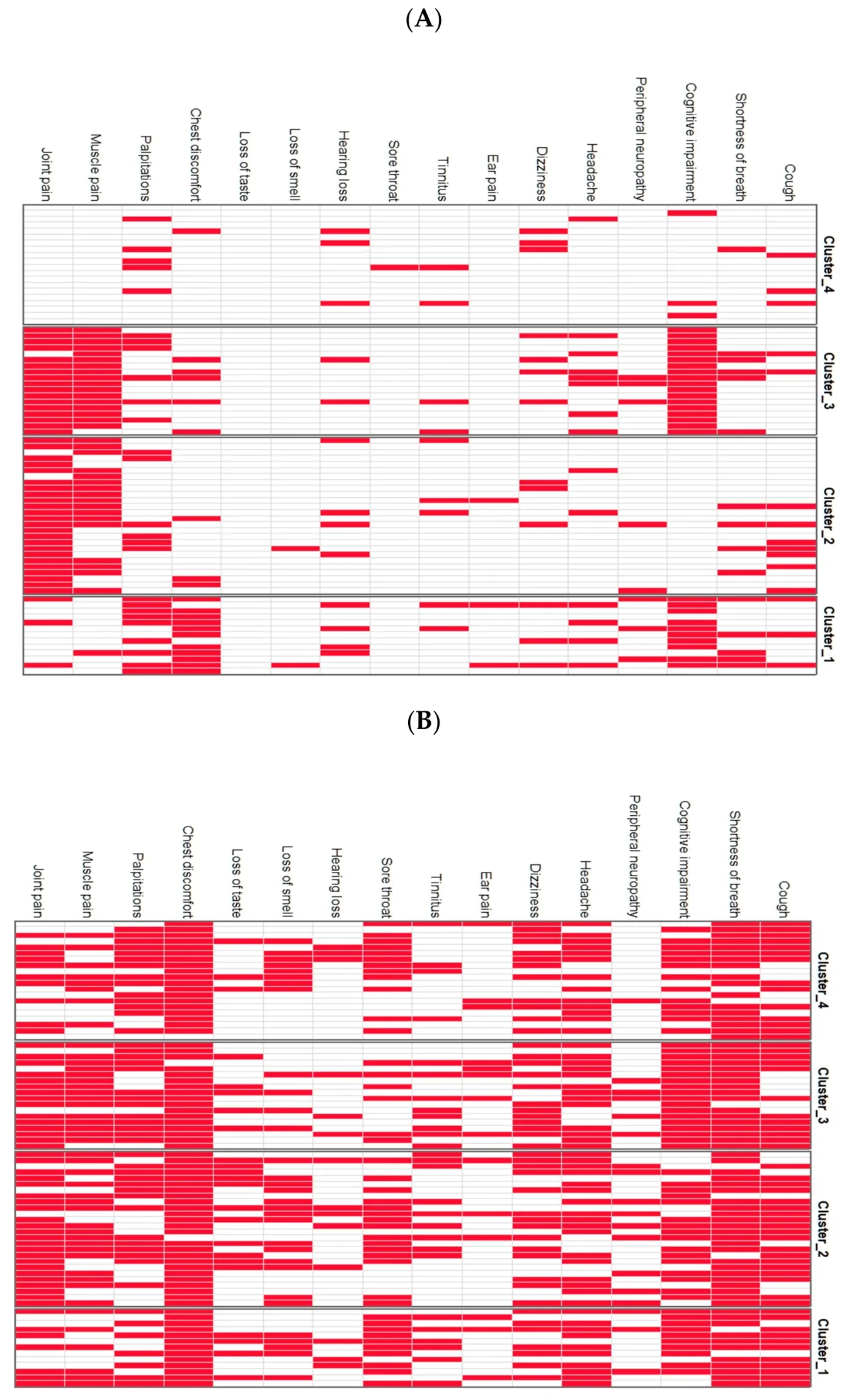
Heatmap visualization of symptom-based long-term PASC profiles (*n* = 77). (**A**) Symptom-based clustering at the 48-month endpoint, demonstrating distinct symptom patterns. (**B**) Retrospective projection of 48-month cluster membership onto the 11-month symptom profiles to demonstrate differences in symptom patterns between the two assessment points. In both panels, columns represent assessed clinical manifestations, rows represent individual participants, red tiles indicate the presence of a symptom, and white tiles denote its absence. PASC: post-acute sequelae of SARS-CoV-2 infection.

**Figure 4:**
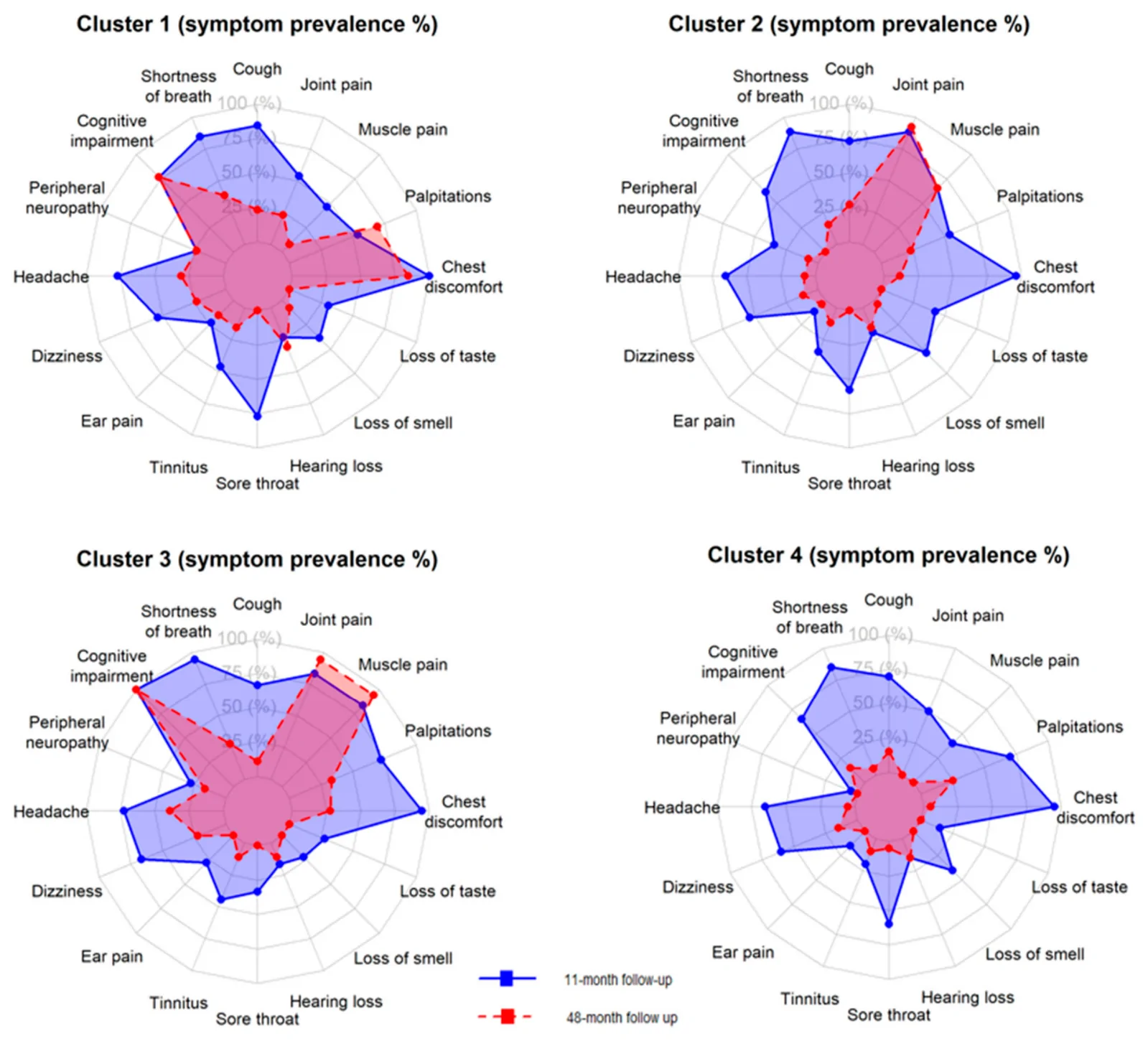
Radar charts depicting symptom prevalence at 11 months (blue) and 48 months (red) within each 48-month-derived PASC cluster. Symptom prevalence is expressed as the percentage of patients reporting each manifestation within each subgroup. The blue polygon represents the early symptom profile at 11 months; the red polygon denotes the endpoint prevalence at 48 months. PASC: post-acute sequelae of SARS-CoV-2 infection.

**Table 1:** Baseline demographic and clinical characteristics of the PASC cohort stratified by 48-month symptom-based cluster.

Variable	Cluster 1 (*n* = 13)	Cluster 2 (*n* = 26)	Cluster 3 (*n* = 18)	Cluster 4 (*n* = 20)
Age, years, median (IQR)	62.0 (14.0)	59.0 (14.0)	56.5 (17.0)	53.5 (14.2)
Range (min–max)	42–73	33–77	28–86	27–68
Sex, *n* (%)				
Male	8 (61.5%)	9 (34.6%)	2 (11.1%)	10 (50.0%)
Female	5 (38.5%)	17 (65.4%)	16 (88.9%)	10 (50.0%)
Comorbidities, *n* (%)				
Asthma	0 (0.0%)	3 (11.5%)	3 (16.7%)	1 (5.0%)
COPD	0 (0.0%)	0 (0.0%)	0 (0.0%)	1 (5.0%)
Allergic rhinitis	0 (0.0%)	3 (11.5%)	0 (0.0%)	5 (25.0%)
Ischaemic heart disease	1 (7.7%)	3 (11.5%)	2 (11.1%)	4 (20.0%)
Heart valve disease	0 (0.0%)	4 (15.4%)	3 (16.7%)	3 (15.0%)
Hypertension	6 (46.2%)	14 (53.8%)	10 (55.6%)	9 (45.0%)
Cardiac arrhythmia	1 (7.7%)	4 (15.4%)	2 (11.1%)	5 (25.0%)
Diabetes mellitus	2 (15.4%)	4 (15.4%)	5 (27.8%)	2 (10.0%)
Smoking history				
Ever-smokers, n (%)	8 (61.5%)	14 (53.8%)	8 (44.4%)	12 (60.0%)
Pack-years, median (IQR)	10.0 (2.5)	10.0 (5.0)	10.0 (7.5)	17.5 (16.2)

**Table 2:** Characteristics and severity of acute COVID-19 infection.

Variable	Cluster 1 (*n* = 13)	Cluster 2 (*n* = 26)	Cluster 3 (*n* = 18)	Cluster 4 (*n* = 20)
Severity (average)	1.78	1.73	1.71	1.73
Respiratory support (*n*)	7	14	9	4
Non-invasive respiratory support (*n*)	7	13	8	3
Invasive respiratory support (*n*)	0	1	1	1
Pulmonary involvement on CT (*n*)	11	19	13	15
Pulmonary involvement in % on CT (median, IQR)	40 (30–60)	30 (20–50)	35 (15–35)	15 (10–25)
ICU (*n*)	2	3	3	1
Vaccination, *n* (%) *	5 (38.46)	12 (46.15)	9 (50.00)	14 (70.00)
Antiviral medication, *n* (%)				
Steroid	7 (53.85)	12 (46.15)	5 (27.78)	4 (20.00)
Favipiravir	11 (84.62)	21 (80.77)	14 (77.78)	17 (85.00)
Remdesivir	4 (30.77)	8 (30.77)	4 (22.22)	4 (20.00)

Note: PASC: post-acute sequelae of COVID-19; IQR: interquartile range; COPD: chronic obstructive pulmonary disease. Categorical variables are expressed as counts (percentages); continuous variables are expressed as medians (interquartile ranges). * The table includes only vaccinations received before the acute COVID-19 infection.

**Table 3:** Longitudinal symptom dynamics from 11 to 48 months evaluated by intra-cluster McNemar analysis.

**Cluster 1**					
**Symptom**	*p*-Value *	*p*-Value **	**Worsening** ***n* (%)**	**Improvement *n* (%)**	**Persistent** ***n* (%; 95% CI)**
Cough	0.0078	0.0200	0 (0.00)	8 (61.54)	3 (27.27; 6.02–60.97)
Shortness of breath	0.0313	0.0606 ^†^	0 (0.00)	6 (46.15)	5 (45.45; 16.75–76.62)
Cognitive impairment	1.0000	1.0000	2 (15.38)	2 (15.38)	8 (80.00; 44.39–97.48)
Peripheral neuropathy	1.0000	1.0000	1 (7.69)	1 (7.69)	2 (66.67; 9.43–99.16)
Headache	0.0703	0.1216	1 ((7.69)	7 (53.85)	3 (30.00; 6.67–65.25)
Dizziness	0.2187	0.3182	1 (7.69)	5 (38.46)	2 (28.57; 3.67–70.96)
Ear pain	1.0000	1.0000	0 (0.00)	1 (7.69)	2 (66.67; 9.43–99.16)
Tinnitus	0.1250	0.1861	0 (0.00)	4 (30.77)	2 (33.33; 4.33–77.72)
Sore throat	0.0019	0.0078	0 (0.00)	10 (76.92)	0 (0.00; 0.00–30.85)
Hearing loss	1.0000	1.0000	1 (7.69)	0 ((0.00)	3 (100.00; 29.24–100.00)
Loss of smell	0.1250	0.1861	0 (0.00)	4 (30.77)	1 (20.00; 0.51–71.64)
Loss of taste	0.1250	0.1861	0 (0.00)	4 (30.77)	0 (0.00; 0.00–60.24)
Chest discomfort	0.5000	0.6154	0 (0.00)	2 (15.38)	11 (84.62; 54.55–98.08)
Palpitations	0.5000	0.6154	2 (15.38)	0 (0.00)	7 (100.00; 59.04–100.00)
Muscle pain	0.1250	0.1861	1 (7.69)	6 (46.15)	0 (0.00; 0.00–45.93)
Joint pain	0.1250	0.1861	0 (0.00)	4 (30.77)	3 (42.86; 9.90–81.59)
**Cluster 2**					
**Symptom**	*p*-Value *	*p*-Value **	**Worsening *n* (%)**	**Improvement** ***n* (%)**	**Persistent** ***n* (%; 95% CI)**
Cough	<0.001	0.0026	0 (0.00)	12 (46.15)	7 (36.84; 16.29–61.64)
Shortness of breath	<0.001	<0.001	0 (0.00)	19 (73.08)	4 (17.39; 4.95–38.78)
Cognitive impairment	<0.001	<0.001	0 (0.00)	16 (61.54)	0 (0.00; 0.00–20.59)
Peripheral neuropathy	0.0156	0.0357	0 (0.00)	7 (26.92)	2 (22.22; 2.81–60.01)
Headache	<0.001	<0.001	0 (0.00)	15 (57.69)	2 (11.76; 1.46–36.44)
Dizziness	0.0074	0.0200	2 (7.69)	13 (50.00)	1 (7.14; 0.18–33.87)
Ear pain	0.0625	0.1143	0 (0.00)	2 (7.69)	1 (33.33; 0.84–90.57)
Tinnitus	0.0313	0.0606 ^†^	0 (0.00)	6 (23.08)	3 (33.33; 7.49–70.07)
Sore throat	<0.001	<0.001	0 (0.00)	15 (57.69)	0 (0.00; 0.00–21.80)
Hearing loss	1.0000	1.0000	2 (7.69)	3 (11.54)	2 (40.00; 5.27–85.34)
Loss of smell	<0.001	0.0020	0 (0.00)	13 (50.00)	1 (7.14; 0.18–33.87)
Loss of taste	<0.001	0.0048	0 (0.00)	11 (43.31)	0 (0.00; 0.00–28.49)
Chest discomfort	<0.001	<0.001	0 (0.00)	22 (84.62)	3 (12.00; 2.55–31.22)
Palpitations	0.0078	0.0200	0 (0.00)	8 (30.77)	6 (42.86; 17.66–71.14)
Muscle pain	1.0000	1.0000	4 (15.38)	4 (15.38)	13 (76.47; 50.10–93.19)
Joint pain	1.0000	1.0000	1 (3.85)	0 80.00)	23 (100.00; 85.18–100.00)
**Cluster 3**					
**Symptom**	*p*-Value *	*p*-Value **	**Worsening *n* (%)**	**Improvement** ***n* (%)**	**Persistent** ***n* (%; 95% CI)**
Cough	0.0064	0.0200	1 (5.56)	11 (61.11)	1 (8.33; 0.21–38.48)
Shortness of breath	<0.001	0.0026	0 (0.00)	12 (66.67)	5 (29.41; 10.31–55.96)
Cognitive impairment	1.0000	1.0000	0 (0.00)	0 (0.00)	18 (100; 81.47–100.00)
Peripheral neuropathy	0.5000	0.6154	0 (0.00)	2 (11.11)	3 (60.00; 14.66–94.73)
Headache	0.0703	0.1216	1 (5.56)	7 (38.89)	6 (46.15; 19.22–74.87)
Dizziness	0.0215	0.0474	1 (5.56)	9 (50.00)	3 (25.00; 5.49–57.19)
Ear pain	0.0625	0.1143	0 (0.00)	5 (27.78)	0 (0.00; 0.00–52.18)
Tinnitus	0.0313	0.0606 ^†^	0 (0.00)	6 (33.33)	2 (25.00; 3.19–65.09)
Sore throat	0.0313	0.0606 ^†^	0 (0.00)	6 (33.33)	0 (0.00; 0.00–45.93)
Hearing loss	1.0000	1.0000	0 (0.00)	1 (5.56)	2 (66.67; 9.43–99.16)
Loss of smell	0.1250	0.1861	0 (0.00)	4 (22.22)	0 (0.00; 0.00–60.24)
Loss of taste	0.0625	0.1143	0 (0.00)	5 (27.78)	0 (0.00; 0.00–52.18)
Chest discomfort	<0.001	0.0026	0 (0.00)	12 (66.67)	5 (29.41; 10.31–55.96)
Palpitations	0.0156	0.0357	0 (0.00)	7 (38.89)	6 (46.15; 19.22–74.87)
Muscle pain	0.5000	0.6154	2 (11.11)	0 (0.00)	15 (100.00; 78.20–100.00)
Joint pain	0.5000	0.6154	2 (11.11)	0 (0.00)	15 (100.00; 78.20–100.00)
**Cluster 4**					
**Symptom**	*p*-Value *	*p*-Value **	**Worsening *n* (%)**	**Improvement** ***n* (%)**	**Persistent** ***n* (%; 95% CI)**
Cough	0.0034	0.0122	1 (5.00)	12 (60.00)	2 (14.29; 1.78–42.81)
Shortness of breath	<0.001	<0.001	0 (0.00)	16 (80.00)	1 (5.88; 0.15–28.69)
Cognitive impairment	0.0019	0.0078	0 (0.00)	10 (50.00)	3 (23.08; 5.04–53.81)
Peripheral neuropathy	1.0000	1.0000	0 (0.00)	1 (5.00;	0 (0.00; 0.00–97.50)
Headache	<0.001	0.0026	0 (0.00)	12 (60.00)	1 (7.69; 0.19–36.03)
Dizziness	0.0117	0.0289	1 (5.00)	10 (50.00)	2 (16.67; 2.09–48.41)
Ear pain	0.2500	0.3478	0 (0.00)	3 (15.00)	0 (0.00; 0.00–70.76)
Tinnitus	0.6250	0.7547	1 (5.00)	3 (15.00)	1 (25.00; 0.63–80.59)
Sore throat	0.0034	0.0122	1 (5.00)	12 (60.00)	0 (0.00; 0.00–26.46)
Hearing loss	1.0000	1.0000	1 (5.00)	1 (5.00)	2 (66.67; 9.43–99.16)
Loss of smell	0.0078	0.0200	0 (0.00)	8 (40.00)	0 (0.00; 0.00–36.94)
Loss of taste	0.2500	0.3478	0 (0.00)	3 (15.00)	0 (0.00; 0.00–70.76
Chest discomfort	<0.001	<0.001	0 (0.00)	18 (90.00)	1 (5.26; 0.13–26.03)
Palpitations	0.0039	0.0131	0 (0.00)	9 (45.00)	5 (35.71; 12.76–64.86)
Muscle pain	0.0078	0.0200	0 (0.00)	8 (40.00)	0 (0.00; 0.00–36.94)
Joint pain	0.0019	0.0078	0 (0.00)	10 (50.00)	0 (0.00; 0.00–30.85)

Note: * raw *p*-value; ** BH-adjusted *p*-value; ^†^ Statistical significance was lost after Benjamini–Hochberg correction (FDR < 0.05).

## Data Availability

The data presented in this study are available on request from the corresponding author.

## Supplementary Materials

The following supporting information can be downloaded at: https://www.mdpi.com/article/10.3390/jcm15197487/s1, Table S1: Symptom prevalence at 11 and 48 months across clinical clusters.

## Abbreviations

The following abbreviations are used in this manuscript:

PASC	post-acute sequelae of SARS-CoV-2 infection
DEKK	Clinical Centre of the University of Debrecen
CT	computed tomography
SD	standard deviation
IQR	interquartile range
COPD	chronic obstructive pulmonary disease
HRCT	high-resolution computed tomography
